# Sexual slavery without borders: trafficking for commercial sexual exploitation in India

**DOI:** 10.1186/1475-9276-7-22

**Published:** 2008-09-25

**Authors:** Christine Joffres, Edward Mills, Michel Joffres, Tinku Khanna, Harleen Walia, Darrin Grund

**Affiliations:** 1 Simon Fraser University, Faculty of Health Sciences, Blusson Hall, Room 11300, 8888 University Drive, Burnaby, B.C. V5A 1S6, Canada; 2 State Coordinator, Bihar Anti-trafficking Resource, Centre Apne Aap Women Worldwide , Jagdish Mills Compound, Forbesganj, Araria, Bihar 841235, India; 3 Technical Support – Child Protection, GOI (MWCD)/UNICEF, 253/A Wing – Shastri Bhavan,, Dr. Rajendra Prasad Marg,, New Delhi:110001, India

## Abstract

Trafficking in women and children is a gross violation of human rights. However, this does not prevent an estimated 800 000 women and children to be trafficked each year across international borders. Eighty per cent of trafficked persons end in forced sex work. India has been identified as one of the Asian countries where trafficking for commercial sexual exploitation has reached alarming levels. While there is a considerable amount of internal trafficking from one state to another or within states, India has also emerged as a international supplier of trafficked women and children to the Gulf States and South East Asia, as well as a destination country for women and girls trafficked for commercial sexual exploitation from Nepal and Bangladesh. Trafficking for commercial sexual exploitation is a highly profitable and low risk business that preys on particularly vulnerable populations. This paper presents an overview of the trafficking of women and girls for sexual exploitation (CSE) in India; identifies the health impacts of CSE; and suggest strategies to respond to trafficking and related issues.

## Introduction

An estimated 800 000 women and children are trafficked each year across international borders, 80% ending in forced sex work [1]. This estimate does not include those trafficked within their own countries or missing children. Human trafficking for commercial sexual exploitation (CSE) is a gross violation of human rights and has been described as a modern form of slavery [2]. The United Nations (UN) estimates that the trafficking of women and children for CSE in Asia has victimized over 30 million people [3]. India has been identified as one of the Asian countries with a severe CSE trafficking problem. The U.S Department of State has put India on the Tier 2 Watch List, for the fourth consecutive year. It has also warned India that it could be downgraded to a Tier 3 category, thereby exposing itself to international sanctions, unless it improves its record on trafficking [1]. This paper presents an overview of the trafficking of women and girls for sexual exploitation (CSE) in India; identifies the health impacts of CSE; and suggest strategies to respond to trafficking and related issues. To our knowledge, this article is the first condensed and comprehensive paper on CSE in India.

For the purpose of this paper, trafficking is defined as 'the act of recruitment, transportation, transfer, harbouring or receipt of a girl or women for the purpose of exploitation' (Palermo Protocol art. 3; Council of Europe Convention art. 4). Underage children are children below 18 years of age.

Some of India's characteristics are described in Table 1.

**Table 1:** India at a glance

Population*:	~1.13 billion	Under 5 mortality rate (2005)**:	74
Population under 14*:	32.1%	Infant mortality rate (under 1) (2005)**	56
Net migration*:	-0.3	Life expectancy at birth (2005)**:	64 yrs.
Total adult literacy rate 2000–2004*:	61	GNI per capita (US$ – 2005)***:	730
Human Development Index*:	127th/177	% of people living in poverty (99-05)***:	29
Gender-related Development Index*:	98^th^/140	Average annual growth GDP (2005)***:	9.2
TI – Corruption Perceptions Index*:	2.8/10	Average annual growth GDP/capita***:	7.7
WEF – Organized crime index*:	5.1/7		

Sources: *: United Nations (Office of Drugs and Crime):

**: Unicef: India:

***: World Bank:

## Methods

A literature review and annotated bibliography were generated through the systematic search of several online databases including Medline, Sociological Abstracts, and Social Sciences Citation Index to locate peer-reviewed literature, as well as Google.com to locate gray literature. Key words describing or related to human trafficking for commercial sexual exploitation and its health impacts, as well as their synonyms, were identified via MeSH (medical subject heading) (e.g., sex trafficking, trafficking of women and girls, female sex workers, female prostitution) and guided the literature search. A manual search of the reference lists of the retrieved documents was also performed. We contacted the publishers or organizations of documents that were not retrievable on the internet or the university library. All together, we reviewed 261 articles. Additionally, we sent this paper to NGOs working with women who have been sex-trafficked in India for their comments and review.

### Overview of trafficking for Commercial Sexual Exploitation (CSE) in India

The clandestine and transnational nature of trafficking and the reluctance of those involved to discuss the topic make accurate assessments of the magnitude of CSE difficult [4]. A recent government commissioned by the Department of Women and Child Development (India) estimated the number of persons trafficked for CSE in India to be around 2.8 million, an increase of 22% from an earlier estimate [5,6]. The majority of trafficked persons are young women or children who have been forced into sex work as a result of poverty, often before they were 18 years old. Published literature further points to an increasing demand for younger children and virgins, partly fuelled by the fear of HIV/AIDS; the emergence of new sources and destinations for trafficked persons; and an increase in the overall sophistication of trafficking networks, many of which are controlled by organized crime syndicates or insurgent factions [3,7,8]. This has been illustrated in Nepal where the traditional trafficking of Nepalese girls to Indian brothels had been taken over by Nepalese rebel groups in order to fund their fight against the state [9].

**Figure 1:**
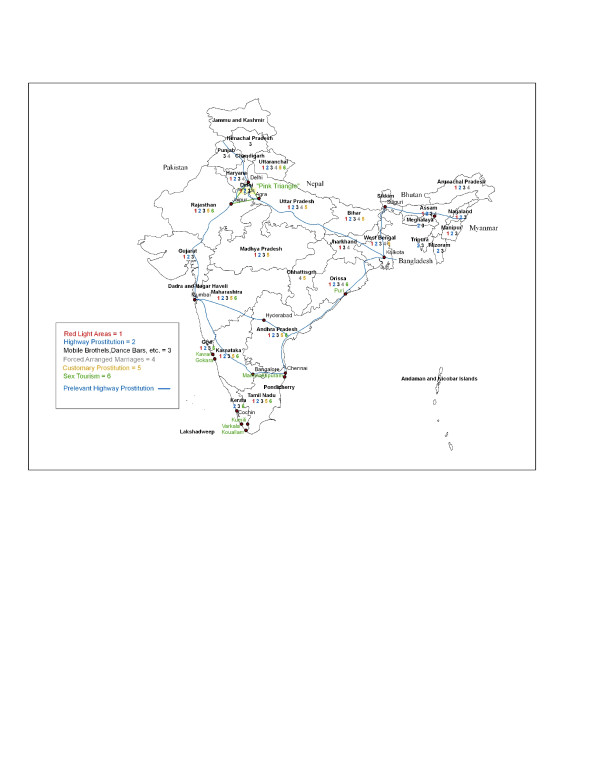
Illustrates the different forms of sexual exploitation in India.

The magnitude of the problem may partly be accounted by the different forms of sexual exploitation in India (Figure 1) [10-20]. The most common form of sex work involves young women and girls from economically deprived and marginalized groups (e.g. Dalits) who have been 'recruited' by brokers, sold to pimps or brothel owners (most of whom are ex-prostitutes), and forced into prostitution. Brokers may be community members known to the victims or the victims' families pretending to help families; agents seeking the help of a local person to approach families and victims; individuals willing to kidnap potential victims; and family members (e.g., parents or husbands). Recruitment strategies include: false promises of employment; approaching debt-bonded families and persuading them to part with their children to pay for their debts; abduction; and arranged marriages whereby young women and underage girls are 'married' to grooms willing to pay poverty-stricken parents a dowry [3,8,10]. Once married (marriage makes this form of trafficking particularly difficult to challenge under the law), wives are either forced into prostitution directly by their husbands or abandoned/divorced and sold to a broker who resells them to a brothel [10,11]. This practice has been documented in Bihar, West Bengal, Chhatisgarh, Orissa, Uttaranchal, and Hyderabad. Common destinations for women and girls forced into 'arranged' marriages include Punjab, Haryana, Uttar Pradesh, and the United Arab Emirates [11,12].

Customary prostitution is also prevalent in India. It includes socially (if not legally) accepted forms of prostitution, i.e., religious and tribal prostitutions [15-17]. Victims of religious prostitution are pre-pubertal girls from scheduled castes (e.g., Devadisi, Jogini, Nailis, Muralis, and Theradiyan) who are dedicated to different deities. After a few years of concubinage with temple priests, they are sold or auctioned to traffickers for CSE. The market value of girls tends to fall after puberty. This form of prostitution is mostly practiced in Karnataka, Maharahstra, and Andhra Pradesh. Tribal prostitution involves girls from different ethnic tribes (e.g, Bedia, Nats) who used to entertain feudal lords. Overtime, many of these tribal communities have been forced to engage in prostitution for economic reasons. Tribal prostitution is prevalent in Andhra Pradesh, Rajasthan, Maharashtra, West Bengal, Chhattisgargh, and Manipur. However, Orissa, Bihar, and Uttaranchal have also emerged as supply states for tribal prostitution. Victims of customary prostitution are generally forced into prostitution at a very young age (9–13) by family members (parents or brothers) who act as agents of the victims [3,10,12]. Approximately 16% of persons forced in the sex trade are in prostitution as a result of customary practices [18].

Another form of sexual exploitation includes sex tourism (ST). ST includes the sexual exploitation of young boys and girls by international and Indian tourists. Street children are particularly vulnerable to this type of sexual exploitation [19,20]. Costs per sexual act vary from 50 to 200 Indian Rs (about 1 to 5 US$) and can reach up to 1000 Rs (about 25 US$) when victims remain with their clients overnight or longer [19]. Sex tourism is prevalent in the 'pink triangle', i.e., the Agra-Delhi-Jaipur belt, as well as south and south-west India: Goa, Maharashtra (Mumbai), Karnataka (notably, in some of the new tourist spots like Gokarna and Karwar), Kerala (in Kovallam, and other new popular destinations such as Cochin, Kumily, and Varkala), in Tamil Nadu (Mammallapuram), and in Orissa (particularly Puri). Himachal Pradesh and Rajasthan are emerging as new destinations for sex tourism. Sex tourism may be facilitated by travel agencies, tour operators, hotels, and associated business. There is also some evidence of young boys being imported from the Gulf countries into Southern India and forced into prostitution [3,8,20].

In the last 15 years, CSE has been characterized by two changes. Females from upper castes are increasingly becoming victims of CSE [6]. Prostitution is no longer primarily confined to traditional brothels and can be found in new venues, such as mobile brothels, dance bars, escort services, friendship clubs, massage parlours, and huts or bath establishments along the national highways (e.g., the Kolkata,-Siliguri-Guwahati-Shillong highway, the Dehli-Kolkata-Chennai-Mumbai highway, the Solapur-Hyderabad highway; the Grand Trunk Road between Bangaldesh and Pakistan) [3,12-15].

### Internal and transnational trafficking for CSE

**Figure 2:**
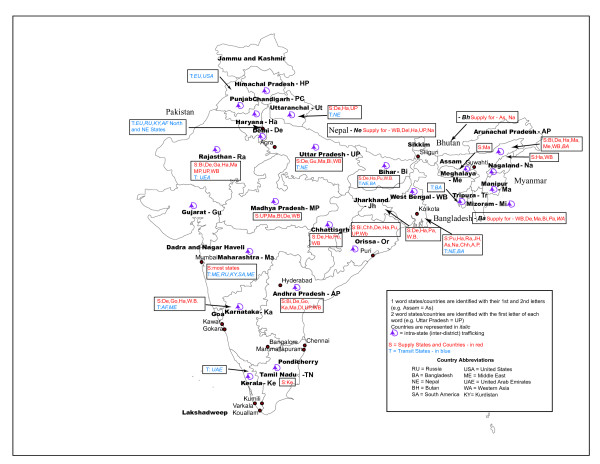
**Presents the states that traffics women and girls for commercial sexual exploitation in India.** Trafficking involves supply (red in the figure), transit (blue in the figure), and/or destination states.

India is a source, transit, and destination country for women and girls trafficked for CSE [3,7,8]. Interstate trafficking represents 89% of trafficking for CSE in India. Figure 2 presents the main supply, transit, and destination states. The biggest supply states include: Andhra Pradesh (16/23 districts affected by trafficking for CSE), Bihar (24/38 districts affected by trafficking), Madhya Pradesh, West Bengal, Karnataka (16/27 districts affected by CSE), Tamil Nadu, Maharashtra, and Uttar Pradesh. They are followed by the states of Orissa, Rajasthan, Assam, and Jharkhand. Bihar, Maharashtra, and Madhya Pradesh have also the dubious distinction of being the states that procure the largest number of minor girls. The biggest buyers of minors include West Bengal and Maharashtra [7,8,12]. Main destinations include Delhi, West Bengal (Kolkata), Maharashtra (Mumbai), Gujurat, Punjab, and Haryana (the last 2 states being particularly popular for 'arranged' marriages). Cities like Mumbia, Dehli, Kolkata, Bangalore, Hyderabab, and Chennai have the largest concentration of prostitutes [10]. Table 2 describes some of the characteristics of brothels in the most prominent red lights areas of India.

**Table 2:** Characteristics of brothels in major red light areas

Mumbai
• Mumbai generates at least $400 million/year in revenue from the estimated 100,000 women/girls serving an average of 6 customers per day; it is also a centre for pedophiles
• Minors (10–14) trafficked for CSE are often kept in cage-like confinements
• Criminal nexus in trafficking is visible
• Kamathipura is the largest brothel area with 20,000 women and girls working in prostitution
Delhi
• More than 20,000 women/girls of different age/groups (many 12–13 years) in 3,000 red light areas.
• 90 brothels at G.B. Road alone with an estimated 4,000 prostitutes
• Majority are kept in slave-like conditions whereby brothel owners take victims' earnings until repayment of the price at which they were bought, which takes 4–8 years

Kolkata
• 29 red light areas, including Sonagachi, Kidderpore, Kalighat, Rambagan, Bowbaza
• Sonagachi has been in existence for at least 150 years. It has 4,000–6,000 sex workers working in 370 brothels servicing about 20,000 clients a day
• Many brothels consist of several rooms divided by curtains into booths, each with a bed.
• Transit point for many girls who are 'initiated' into the business before being resold to other brothels

Goa
• The state for sex tourism and pedophiles (Indians and foreigners)
• Red Light areas are smaller than in Kolkota or Mumbai but share many of the same characteristics.
• Baina has about 3,000 prostitutes.
• Most women are debt-bonded and released after 2–3 years receiving only 20% of their income.

Sources: Asian Development Bank, 2002, 2003 [6,7]; Terres des Hommes, 2005 [21]

Trafficking from neighbouring countries into India account for about 10% of trafficking for CSE. Bangladesh and Nepal are the biggest suppliers, respectively accounting for 2.17% and 2.6% of the international traffic for CSE into India [3,7,8]. An estimated 10,000–20,000 Bangladeshi women and girls are trafficked every year into India. Total estimates of Bangladeshi persons trafficked for CSE into India vary between 200 000–300 000. Trafficked victims are initially kept in West Bengal, Assam, Orissa, or Tripura. There, they are 'graded' according to their age, beauty (e.g., light or dark skin), and sexual status (e.g., virgin), initiated into commercial sexual customs, and sent to new destinations, including New Delhi, Agra, Mumbai, Kolkota, Goa, or Pakistan (Karichi and Lahore).

Estimates of Nepali persons trafficked into India vary from 100 000 to 200 000 [6]. Human Rights Watch (2005) estimated that 6,000–10,000 Nepalese were trafficked into India annually. Other studies have suggested similar numbers (5,000–11,000) [21-24]. Seventy per cent of trafficked victims, most of them under 16, come from ethnic minority groups (e.g., Tamang, Gurung, Magar, and Sherpa) who live in remote hill villages or poor border communities. Trafficked victims are sold to brokers from amounts as small as 200 Nepali Rs. 200 (US$4.00) who then deliver them to Indian brothels for amounts ranging from 15,999 to 40,000 Rs (US$ 500–1300) [22,24]. India is also a transit country for Nepalese and Bangladeshi women trafficked to Pakistan, Western Asia, and the Middle East and for women trafficked from the Russian Federation to Thailand [25].

Table 3 shows the data available on women trafficked from Bangladesh and Nepal into Indian brothels. It does not include non-brothel based prostitution or prostitution into small urban and rural red light areas. The literature also indicates that India is a destination country for women trafficked from Bhutan, Myanmar, Kyrgyzstan, Pakistan, Europe, Russia, and Thailand [12,25].

**Table 3:** Estimates of the number of women trafficked from Bangladesh and Nepal into Indian brothels

No. of trafficked women	Nationality	Destination	Sources
70% of 1,000 to 10,000	Bangladeshi	Kolkota (last 5 years	Asian Bank Development (ADB) [7,8]
800	Bangladeshi	Kolkota (1990–92)	ADB [7,8]
30,000	Bangladeshi	Kolkota	ADB [7,8];Coalition Against Trafficking in Women (CATW):
2,000	Bangladeshi	Various cities	ADB [7,8]; CATW – Asia Pacific: ;
10,000	Bangladeshi	Mumbai, Goa	ADB [7,8]; CATW:
200,000	Nepalese	-	
27,000	Bangladeshi	-	ADB [7,8]
300,000	Bangladeshi	Various cities	Centre for Health and Population Research:
45,000	Nepalese	Mumbai	ADB [7,8]; Child Workers in Nepal Concerned Center (CWIN) (year not provided)
35,000	Nepalese	Kolkata	ADB [7,8]; CWIN (year not provided)
20,000	Nepalese	Delhi	ADB [7,8]; CWIN (year not provided)

Data on India as an international supply country are scarce [7]. However, evidence suggests that children from Karnataka, Andhra Pradesh, and Madhya Pradesh are trafficked to the Gulf States, England, Korea, and the Philippines for CSE [3]. As well, the United Nations reported trafficking for CSE from India to Western Asia (the most prevalent), Kenya, The United Arab Emirates, The United States of America, and to a lesser extent to Bahrain, Bhutan, Canada, France, Germany, Kuwait, Malaysia, Netherlands, Pakistan, Saudi Arabia, Singapore, Thailand, Turkey, The United Kingdom, and The United Republic of Tanzania [25].

### Factors contributing to trafficking for CSE

Trafficking for CSE is a demand-driven phenomenon, facilitated by traffickers, who find trafficking highly profitable and low risk, and the availability of vulnerable populations. Vulnerability results from a range of inter-related economic, social, political, and familial factors (e.g., poverty, lack of sustainable livelihoods, structural inequities) and creates the supply needed by traffickers to meet the nature of the demand. In most cases, trafficking results from the interface of multiple risk factors [2,3,7,8,10,19,20,26-34]. For example, trafficking in Manipur has been fueled by years of civil unrest, the presence of armed forces, drug trafficking from neighbouring Myanmar, and poverty. Factors that facilitate trafficking for CSE are included in table 4.

**Table 4:** Factors contributing to increased vulnerability to trafficking for CSE

Economic factors
• Poverty: families unable to meet basic needs, female-headed households, families without any assets (e.g., due to long term unemployment, under-employment, sudden economic shocks), indebted families from poor states (e.g., Bihar, Uttar Pradesh, Karnataka, Orissa, Rajasthan, Assam, etc.) [2,3,7,8,10]
• Unemployment and migration. Lack of employment opportunities, particularly in rural areas, force individuals or families to migrate to seemingly better places and make them more vulnerable to trafficking (e.g., Orissa, Bihar) [2,3,7,8,10,27]
• Income disparities between rural and urban areas, combined with a desire for a better life[3,7,8,10]
• Growth of tourism in specific areas (e.g., Goa, Kerala, Rajasthan) [19,20]
• Trafficking for CSE has proven to be a low risk and highly lucrative business [3,7,8]
• Globalisation (e.g., Bata has undermined the Regar community in Rajasthan when it started selling shoes in India, as well as recent macro-level agricultural reforms) [28,29]
Environmental factors (resulting in long-term lack of sustainable livelihood) [2,3,7,8]
• Drought (e.g., Rajasthan)
• Cyclones (e.g., in The Sundarbans in Bangladesh) and floods
• The 2004 tsunami (e.g., coastal Tamil Nadu, Andhra Pradesh, & Orissa were particularly affected)
• The closure of sick tea gardens and subsequent layoffs around Darjeeling in West Bengal has resulted in starvation deaths, the suicides of women and children, and women and children being forced into exploitative forms of work and trafficking[30]

Social/cultural factors [2,3,7,8,10,12]
• Tolerance of domestic violence and lack of respect for human rights, particularly women's and children's rights, which push victims to opt out of particularly abusive situations without economic recourses [31]
• Caste-related discrimination that deprives specific groups (e.g., scheduled castes such as the Dalits) of their basic rights (e.g., access to water or medical care) [32]
• Customary prostitution (see Figure 1).
• Arranged/coerced early marriages & dowries (Figure 1)
• Gender discrimination (women seen as a burden on families; low education levels for females, and few or no economic opportunities for females).
• Victimization and stigmatization of divorced, abandoned, and raped women and girls, and/or women and girls who are illegal immigrants in India (e.g., Nepalese and Bangladeshi)
• Beliefs that sex with virgins will cure STDs and sexual dysfunctions.

Governance issues [2,3,7,8,12]
• Wars, civil conflicts, strong presence of armed forces, drug trafficking (Nepalese women and girls are fleeing Nepal to avoid Maoist pressures to join military training or be recruited as child soldiers; Indian States bordering Myanmar: Manipur, Nagaland, and Mizoram) [26]
• Insufficient pro-poor policies and initiatives
• Laws which ignore exploitation of children by their own families
• Unsafe migration, porous borders (between Nepal and Indian and between Bangladesh and India) [33]
• Legislation and enforcement procedures that are inadequate to deter trafficking and bring traffickers to justice, coupled with corruption (see second section: corruption index) [34]

Micro/familial factors [2,3,7,8,10]
• Females from indebted, poverty stricken families
• Single women (unmarried, abandoned, divorced, widowed, rape victims) with or without children
• Adolescent girls/children, particularly children from families where abuse/neglect is prevalent or families in crisis (caused by war, civil unrest, or environmental catastrophes)
• Female migrants, alone or with families
• Females coerced into early marriage (frequent in Chhattisgarh, Haryana, Jharkhand, Bihar, Orissa, and Assam)
• Children of trafficked victims
• Street children (e.g., Goa, Kerala)

### Commercial sexual exploitation and HIV/AIDS

There is little evidence on the morbidity and mortality of persons engaged in commercial sex. Overall, adult HIV prevalence in India is 0.36% (2–3.1 million people infected) [35-39], with 85% of HIV transmitted via heterosexual contacts, except in the northeastern states (Nagaland, Manipur, and Mizoram) where the primary mode of transmission is injection drug use [36]. Estimated HIV prevalence is greater among males (0.43%) than among females (0.29%) [39]. HIV in India continues to be primarily concentrated in high risk populations (i.e., females or males in prostitution and injecting drug users). Estimated adult HIV prevalence was greater than 1% in Manipur (1.67%) and Nagaland (1.26%) in 2006 [39]. HIV prevalence among women in prostitution (WP) in 2003, 2004, 2005, and 2006 was respectively 10.3%, 9.4%, 8.4%, and 5.4 [35-39]. In 2006, HIV among WP was very high in Nagaland (16.4%), followed by Maharashtra (12.8%), Manipur (11.6%), Mizoram (10.4%), Karnataka (9.6%), and Andhra Pradesh (8.8%). Overall, 8 states had greater than 5% HIV prevalence among WP, while 9 states had HIV prevalence between 1 and 5% [39]. Figure 1 shows HIV prevalence among women in prostitution (WP) from 2002 to 2006 in specific states [36-40].

Other studies on specific sub-populations found higher HIV prevalence rates than the ones previously mentioned. For example, a recent study on 287 repatriated Nepalese who had been trafficked in Indian brothels indicated that 38% of the study population was HIV+ [41]. In other studies, HIV prevalence among WP ranged between 43 to 54% of the study population [42-44]. However, the higher HIV prevalence rates in these studies may be due to the small sample size or the selected sample (e.g., WP attending STI clinics).

The aggregate data mask the heterogeneity of the epidemic. This heterogeneity reflects the influence of multiple factors, such as the types of prostitution in different states (e.g., brothel versus non-brothel based), the age at which trafficked persons had their first sexual contacts and/or were forced into CSE, the clients (e.g., regular vs. one-time clients) and broader contextual variables (e.g., the ability of regional and local health care systems to prevent and respond to STIs). HIV prevalence among WP was 45% and 26%, respectively in Mumbai (Maharashtra), and Mysore (Karnataka), and 13%, 27% and 44% in Kolkata, Siliguri, and Panjipara in West Bengal in 2004–2005 [36-40]. However, there are rural districts (e.g., in Rajasthan and Karnataka) where the epidemic is just as advanced as in urban areas [45]. Furthermore, younger WP (25 years of age or less) are more at risk of acquiring STIs than older ones [41,46]. Higher infection rates among younger WP may be associated with the integrity of the genital lining when they are subjected to repeated trauma during sexual intercourse, facilitating higher HIV transmission. Additionally, younger WP may find it more difficult to negotiate condom use with their clients, leading to unprotected sex [41,46].

A limited number of studies have looked at STIs other than HIV among WP (Table 5) [37,42,43,47-53]. These studies (N sizes ranging from 118 to 5574) also suggest that 48–80% of WP have symptoms of STIs. Most common symptoms include vaginal discharge (13–83%), vaginal warts (3–14%), vaginal ulcer (4–25%), Trichomonas (3–40%), Candidiasis (9%), and Gardenella (3%). Overall, brothel-based WP reported less signs of STIs than non-brothel-based WP, except in one study [54]. Interestingly, this study also found that home-based WP were less at risk of acquiring HIV than brothel- or street-based WP.

**Table 5:** Prevalence of STIs other than HIV among WP

Location	N size	Syphilis % of pop	Gonorrhea %	Hepatitis-B %	Chlamydia %
Kolkata [47]	867	26	34		
Ahemedabad [48]	314	24	19		17
Raipur [49]	60	23		8	
Pune [50]	79			69	
Surat [51]	118	23	17		8.5
Kolkata [52]	168	17			

Inconsistent condom use may partly explain STI prevalence. About 50% of the 5574 WP who participated in a national survey used a condom every time with all their *paying *costumers during the 30 days preceding the survey. Consistent condom use with *paying *clients was lower than the national average in Nagaland and Mizoram (23%), Bihar (24%), Assam (27%), Haryana (28%), Rajasthan (34%), and higher than the national average in Kerala (74%), Maharashtra (72.5%), Goa (69%), Delhi (63%), and Madhya Pradesh and Orissa (65%). Brothel-based WP reported more consistent condom use than non-brothel-based WP (57 vs. 46%). However, overall only 20% of WP reported consistent condom use with *non-paying *partners during the 30 days preceding the survey, with the exception of WP in Delhi, Manipur, Karnataka, and Orissa where consistent condom use with *non-paying *partners was higher than the national average. Less than 10% of WP in West Bengal, Bihar, Haryana, Maharashtra, and Punjab reported using condoms consistently with non-paying partners. Consistent condom use with first-time clients was also generally lower than with regular customers. Furthermore, while the national survey indicated that over 70% of WP insisted on using condoms with their clients, only 38% refused to have sex with clients who objected to using condoms. This number is somewhat alarming since 45% of the WP's clients (N = 5684) reported having more than one STI symptoms in the 12 months that preceded the national survey. Also alarming is the fact that only 6.8% of the WP's clients reported using condoms consistently with their regular or lifetime partners [37].

WP's reasons for not using condoms consistently included clients' and partners' objection, complexity of condom negotiation under certain circumstances (e.g., with drunk clients, pimps or the police), lack of access to free or purchased condoms, lack of financial resources to buy condoms, lack of privacy in stores when buying condoms, and social stigma associated with condom purchase [37,55,56]. However, recent studies have highlighted more fundamental issues [57,58]. Lack of funding has hindered the National AIDS Control Organization (NACO) in rolling out an adequate number of interventions targeted at WP. As a result, interventions only reached a small proportion of high-risk individuals, even though the number of WP exposed to STI information/education has steadily increased over the years. Furthermore, the rigidity of financing mechanisms (e.g., USAIDS only fund programs promoting abstinence and condom use but does not fund condom procurement), a lack of strategic planning, a top-down approach to targeted interventions that failed to include the needs, experiences, and perspectives of those working at ground level, including WP, programs that narrowly focused on behavioural changes to the detriment of initiatives focusing on life-skill development and human rights issues have undermined the overall success of condom use interventions. Too often, prevention messages were framed in a moralistic approach promoting abstinence and neglecting the reality that WP may be not able to leave prostitution. Overt and covert discrimination against WP from district hospital personnel and other healthcare workers also prevented WP from seeking necessary health care [59]. Discrimination strategies included denial or delay of treatment, early dismissal, HIV test without WP's consent, and breach of confidentiality re. test results [58,60]. The national surveillance survey highlighted that 13.5% of its sample of over 5500 participants did not seek any treatment when they had STI symptoms, while 16% took home-based remedies and another 5% borrowed prescriptions from friends based on self-diagnosis of symptoms [37]. Further compounding these issues, NGO outreach workers have described a repeated pattern of harassment from the police, including unjustified arrests and coercion, as a major challenge to HIV/AIDs prevention [58].

Although we did not find data on diseases other than STIs among WP, it is likely that the prevalence of chronic diseases among WP is high. These would include pelvic inflammatory disease, chronic pelvic pain, ectopic pregnancy, chronic liver disease and hepatocarcinomas secondary to hepatitis, COPD, asthma and bronchiolitis due to the living conditions, highly polluted environments and moldy dwellings with poor air quality and tobacco smoke. Chronic digestive problems and nutritional deficiencies due to poor nutrition can also be expected. Furthermore, the experience of one of us (H. V.) who has worked as a counselor with rescued victims of CSE for 12 years in India suggests that victims of CSE also suffer from post-traumatic syndrome, severe depression, feelings of helplessness and hopelessness. This has been supported by recent findings on trafficked women [61,62].

## Conclusion – the way forward

The number of women and children trafficked for CSE in India is large [5]. Trafficking in India has become an international business and, unless stringent action is taken, is unlikely to slow down, given the enormous potential profits for organized crime syndicates and independent traffickers. Women and children have a right to be protected from any forms of trafficking and to be treated with dignity. This requires a comprehensive anti-human trafficking strategy embedded in a human rights approach since violations of human rights are both the cause and consequence of human trafficking. Such as approach requires the systematic development and implementation of policies and programmes that address the socio-economic, political, environmental, and cultural factors that increase vulnerability to trafficking at the local, regional, state, national, and international levels [58,63]. India's recent effort toward the harmonization of the anti-trafficking legal framework in India, Nepal and Bangladesh and its new/revised Child Marriage Prohibition Act (2007) are steps into that direction. However, accrued efforts towards the promotion of gender equality in the family, community, and society at large, the facilitation of women's economic empowerment (via job training, job creation and income-generation schemes) and women's ownership and control of productive resources, the development of wider social welfare networks, the enforcement of safe migration policies, and a zero-tolerance level for corruption are also needed. For example, a wider number of border guards, police, and health practitioners should receive specific training related to trafficking.

As many others, we suggest strengthening the Immoral Trafficking Prevention Act, India's legal response to trafficking, in accordance to the United Nations Protocol to Prevent, Suppress and Punish Trafficking in persons, Especially Women and Children (2000) and the UN Recommended Principles and Guidelines on Human Rights and Human Trafficking (2002). Comprehensive anti-trafficking policies not only address the root causes of both supply of and demand for trafficked persons, but they also include the protection of and assistance to trafficked persons, appropriate enforcement mechanisms, and adequate sanctions against traffickers. Protection and assistance to the victims should include compensation for victims of trafficking; necessary medical care; provision of free legal assistance; and the rehabilitation and reintegration of rescued victims in consultation with trafficked victims and countries of origin.

With respect to health, a human rights approach posits that women in prostitution have the same rights to health and safe environments as any other human beings. It requires that STI prevention move away from stereotypical and moralistic notions of prostitution to a broader understanding of the complex nature of human trafficking for CSE; be sensitive to and inclusive of the experiences and perspectives of WP; and extend the locus of responsibility for HIV/AIDS prevention from WP to those participating in or facilitating this business, namely clients, regular partners, the families of WP, brothel owners, pimps, and law enforcement. Interventions also need to address the stigmatization and discrimination against WP at all levels of society (e.g., media, law enforcement, health care, communities), as exemplified by the Sonagachi project launched in Kolkata in 1992 [63,64]. Harassment and ostracism drive epidemics underground and undermine the reach and effectiveness of prevention efforts. This means not only improving the quality of STI interventions for vulnerable women and their partners but also increasing STI monitoring, prevention, and intervention efforts at male sexual workers and men having sex with men. In the context of the global AIDS epidemic, sex between men is significant because it may involve anal sex – a practice that carries a higher risk of HIV transmission than vaginal sex, when no protection is used. Evidence suggests that more people become infected with HIV through male-male sex than via any other transmission route. In India, recent estimations of HIV prevalence among MSM varied from 6.41 to 11.8% [65,66]. However, this group has largely been ignored due to the reluctance of the government to acknowledge and monitor MSM. In fact, sex between men is considered a criminal act in India. As a result, MSM often hide their same-sex relations from friends and families. Many have wives, or have sex with women as well as men, transmitting HIV to their female partners if they are infected. The impact that HIV may have on MSM is therefore not an isolated problem, but one that is linked to the country's wider HIV epidemics, and one that needs t be addressed with greater efforts.

## Competing interests

The authors declare that they have no competing interests.

## Authors' contributions

CJ conceptualized and drafted the paper. EM, MJ, TK, and HW provided substantive comments on the text. DG developed the figures. All authors read and approved the final manuscript.
